# Functions of *Pugionium cornutum* (L.) Gaertn Extracts: Investigating the Mechanism of Gastroparesis Amelioration from the Perspective of the Gut Microbiota and Its Metabolites

**DOI:** 10.3390/foods14162800

**Published:** 2025-08-12

**Authors:** Yangzu Gao, Haoyu Li, Qian Wu, Bang Chen, Kangzhen Xu, Cong Li, Yehua Shen

**Affiliations:** 1 College of Chemical Engineering, Northwest University, Xi’an 710127, China; 2 Key Laboratory of Synthetic and Natural Functional Molecule of the Ministry of Education, National Demonstration Center for Experimental Chemistry Education, College of Chemistry and Materials Science, Northwest University, Xi’an 710127, Chinabangchen@nwu.edu.cn (B.C.); licong@nuw.edu.cn (C.L.); 3 Shaanxi Key Laboratory of Chemical Reaction Engineering, College of Chemistry and Chemical Engineering, Yan’an University, Yan’an 716000, China

**Keywords:** *Pugionium*, gastroparesis, metabolites, gut microbiota, active ingredients

## Abstract

The functional exploration of natural foods, coupled with the increasing prevalence of gastrointestinal motility disorders and the associated therapeutic challenges, has generated significant interest in this field. This study aims to investigate the ameliorative effects of the extract from *Pugionium cornutum* (L.) Gaertn (EAEPC), a traditional edible vegetable in northwest China’s desert region, on atropine-induced gastroparesis in mice, as well as to elucidate its mechanism in terms of the gut microbiota and major metabolites. The findings indicate that EAEPC effectively reduces the rate of pigment residual in the stomach while shortening the gastrointestinal transit time and alleviating other symptoms associated with atropine-induced gastroparesis. These effects may be mediated through modulation of the expression levels of major intestinal metabolites, such as short-chain fatty acids (SCFAs), bile acids (BAs), and L-tryptophan, alongside remodeling of both the diversity and relative abundance of the gut microbiota. Furthermore, correlation analyses were conducted on significantly altered strains and metabolites to clarify their interactions. Moreover, the chemical constituents of EAEPC were identified by UPLC-Q-TOF-MS/MS, and the key active components responsible for improving gastroparesis were predicted through network pharmacology approaches and validated experimentally. These results provide a foundation for further research into the functions of *Pugionium* and offer scientific support for developing natural plant-based strategies aimed at treating gastrointestinal motility disorders.

## 1. Introduction

Up to 40% of the general population suffers from gastrointestinal motility disorders due to the pace of modern life and changes in dietary habits [1,2]. The main manifestations of gastrointestinal motility disorders are functional dyspepsia, gastroparesis, and constipation [3]. Among them, gastroparesis is a disease characterized by weakened gastric motility, delayed gastric emptying, and disordered gastric rhythm but without mechanical obstruction [4]. Although the precise epidemiology of this disease remains unknown [5], it is clear that its incidence rate gradually increases with age and is higher in women than in men. Currently, the main treatment for gastroparesis relies on chemical drugs; however, long-term use of these drugs may result in drug dependence and serious adverse effects such as headaches, tachycardia/palpitations, diarrhea, and hypotension.

Seeking healthier, safer, and more effective alternatives has become a new research direction, and alternative treatments emphasize dietary management to ensure a sufficient intake of dietary fiber and fluids [6]. Multiple studies have shown that food containing dietary fiber, active chemicals, prebiotics, etc., may be beneficial to gastrointestinal motility. Compared to dietary fiber, chemically active components contained in foods have received more attention for their functional activities, such as antioxidant, antimicrobial, anti-aging, and intestinal microenvironmental benefits; thus, it is promising to identify and screen substances from foods that can improve gastrointestinal motility and clarify their possible mechanisms for research and application. *Pugionium cornutum* (L.) Gaertn (abbreviated as *Pugionium*) is an edible wild vegetable with a distinctive pungent smell that is widely found in the desert regions of northern China [7]. Previous research has shown that *Pugionium* is not only rich in various amino acids and vitamin C with good nutritional value [8] but also promotes gastrointestinal motility function and shortens the gastrointestinal transit time in healthy mice [9]. However, its improvement in diseases related to gastrointestinal disorders has not been studied, and the underlying mechanisms are not clear.

Studies have shown that extracts of some edible plants, such as *Sargassum plagiophyllum* extract pretreatment, increased the frequency of gut contractions, leading to a reduced gut transit time. This is associated with an increase in Bifidobacteria in the cecal contents [10]. Aqueous extracts of *Aurantii Fructus Immaturus* and *Magnoliae officinalis Cortex* significantly increased the gastrointestinal transit time and gut propulsion rate in mice with slow-transit constipation, which may be related to the increased diversity and abundance of gut microbiota, in particular, bacteria that produce short-chain fatty acids (SCFAs) [11]. Mao Jian Green Tea ethanol extract significantly increased gastric emptying and the small intestinal propulsion rate in rats with intestinal stress syndrome, which was related to the increased diversity of gut microbiota and the increased abundance of 5-hydroxytryptamine (5-HT)-related bacteria [12]. These findings suggest a possible mechanism of action: functional foods can affect gastrointestinal motility by regulating the gut microbiota. The composition of the gut microbiota and its metabolites has been proven to affect gastrointestinal peristalsis [13,14], and its main metabolites, SCFAs, can act on appropriate receptors to regulate gastrointestinal motility [15].

This research evaluated through animal experiments whether the extract of *Pugionium* could improve gastroparesis caused by atropine, explored its mechanism of action from the perspective of the gut microbiota and its main metabolites, and explained the relationship between the main changed strains and metabolites through relevant analyses. It also predicted its main active ingredients based on network pharmacology and conducted experimental verification. The research results provide a theoretical basis for the development of functional foods derived from *Pugionium cornutum* (L.) Gaertn.

## 2. Materials and Methods

### 2.1. Materials

The positive control agent, Mosapride citrate (tablets), was purchased from YaBao Pharmaceutical, China. Atropine sulfate was obtained from Guizhou JinYu Pharmaceutical, China. L-tryptophan, tryptamine, cholic acid, chenodeoxycholic acid, deoxycholic acid, chloramphenicol, lithocholic acid, 4-hydroxybenzylamine, and other standards were purchased from Shanghai Aladdin Biochemical Technology Co., Ltd. (Shanghai, China). The purities of all the reference standards were greater than 98%.

### 2.2. Preparation of Extract Samples

Fresh plants (ground part) of *Pugionium* were purchased from the local market in Yuyang District, Yulin City, Shaanxi Province, naturally dried and sealed for storage. The extract of *Pugionium* was first prepared by placing 2500 g of dried *Pugionium* in a zipped gauze bag and completely immersing it in a container containing 95% ethanol (*w*:*w* = 1:10); the solution was collected after 48 h. The filtrate was concentrated three times to obtain the ethanolic extract of *Pugionium* (EPC). The EPC was completely dissolved and added to a dispensing funnel; petroleum ether, dichloromethane, and ethyl acetate were added sequentially for extraction; the ethyl acetate extract was collected, concentrated, and dried to obtain the ethyl acetate extract of *Pugionium* (EAEPC).

### 2.3. Determination of the Effect of Improving Gastroparesis

In total, 120 female Kunming mice (20–25 g) were acquired from the Experimental Animal Center of the Air Force Military Medical University (SCXK (Shaanxi) 2023-001). The mice were housed under a temperature of 25 ± 2 °C and a relative humidity of 50 ± 5%, with a 12 h light–dark cycle. The experimental methodology was conducted according to the relevant guidelines and approved by the Northwest University Ethics Committee for Animal Experiments (license No. NWU-AWC-20231101M). After the acclimatization period (7 days), the 120 mice were randomly assigned to six groups: control group (CG), model group (intragastric administration of atropine, AG, 2 mg/kg), and EAEPC high-, medium-, and low-concentration groups (EAEPC-H, EAEPC-M, and EAEPC-L at 10/20/40 g (crude drug)/kg; equal to 2.4/4.8/9.6 mg/kg, respectively) (n = 20 per group).

According to the methods in [8,16,17], the model and intervention groups were injected intraperitoneally with atropine (20 mL/kg) for 7 days to construct the model, while the CG was given the same amount of normal saline. Then, each concentration intervention group was given 20 mL/kg/day of extract for 14 consecutive days, while the CG and AG were administered equal amounts of saline. On the 14th day after administration, 10 mice in each group were randomly selected after fasting for 16 h. The gastrointestinal transit time (GIT), fecal number, and quality of mice within 8 h were observed and recorded. The other 10 mice were anesthetized using isoflurane and used to determine the rate of pigment residual in the stomach. The colonic contents (feces) were collected and kept in a −80 °C freezer.

### 2.4. Exploration Mechanism

#### 2.4.1. Targeted Metabolomics Analysis of Major Metabolites

Targeted metabolomics was determined by high-performance liquid chromatography–mass spectrometry 9030 (Shimadzu, Japan), paired with a column of ZORBAX SB-C18 (4.6 mm × 250 mm, 5 μm). In this experiment, the mobile phases used were A (water, with 0.1% formic acid) and B (acetonitrile, with 0.1% formic acid); the column temperature was 40 °C; and the injection volume was 6 μL. The ion source was an electrospray ion source (ESI), and the parameters were as follows: interface voltage, 4.5 kV; DL temperature, 250 °C; desolvation temperature, 526 °C; interface temperature, 300 °C; heater flow rate, 10.0 L/min; nebulizing gas flow rate, 3.0 L/min; and drying gas flow rate, 10.0 L/min. Afterwards, all processed supernatants were filtered through a 0.22 μm organic membrane before quantification. Multi-reaction monitoring (MRM) scanning mode conditions and mass spectral parameters are shown in Table S1.

SCFAs were determined following the method in [18]. In total, 50 mg of lyophilized feces and 1 mL of acetonitrile were mixed in a centrifuge tube. After mixing, the samples were extracted by low-temperature ultrasonication for 15 min, and the supernatant was collected by centrifugation (20,000× *g*, 10 min, 4 °C). To transfer 120 μL of supernatant into another centrifuge tube, 60 μL of 3-NPH·HCl (200 mmol/L) and 60 μL of EDC·HCl (120 mmol/L) were added and incubated at 40 °C for 30 min. The resulting solution was diluted to 1 mL with a 50% acetonitrile solution and filtered for measurement. The mobile flow rate was 1 mL/min, and the elution conditions were set to 30% B-phase isocratic elution; then, after 20 min, the controller stopped. MRM scanning mode in negative-ion mode was used.

Free bile acids (BAs) were determined following the methods in [19,20]. In total, 60 mg of lyophilized feces was loaded into a centrifuge tube with 600 µL of pre-cooled methanol (containing 5 µg/mL chloramphenicol). Ultrasound-assisted extraction was performed at a low temperature for 20 min, followed by centrifugation (20,000× *g*, 10 min, 4 °C) and the collection of the supernatant. The mobile flow rate was 0.4 mL/min, and elution conditions (A:B) were as follows: 0.01 min, 90:10; 2 min, 90:10; 8 min, 30:70; 15 min, 10:90; 20 min, 0:100; 23 min, 0:100; 25 min, 90:10; and 30 min, the controller stopped. The scanning mode was MRM in negative-ion mode.

L-tryptophan and its metabolites were determined following the method in [21]. In total, 60 mg of lyophilized feces, 100 μL water (containing 0.2% formic acid), and 300 μL acetonitrile solution (containing 500 ng/mL 4-hydroxybenzylamine and 2% formic acid) were sequentially placed in a centrifuge tube. The mixture was vortexed and subjected to ultrasound-assisted extraction at 4 °C for 10 min and then centrifuged (20,000× *g*, 10 min, 4 °C) to collect the supernatant. The mobile flow rate was 0.4 mL/min, and the elution conditions (A:B) were as follows: 0.01 min, 97:3; 10 min, 55:45; 13 min, 55:45; 18 min, 15:85; 20 min, 97:3; and 25 min, the controller stopped. The scanning mode was MRM in positive-ion mode.

##### 2.4.2. 16S rRNA High-Throughput Sequencing for Gut Microbiota

Microbial DNA was used as a template to amplify the hypervariable regions (V3-V4) of the 16S rRNA gene in preparation for sequencing. Sample amplicons were sequenced on an Illumina HiSeq platform (Illumina, San Diego, CA, USA), and 250 bp paired-end reads were generated. Most of the test procedures were performed by Majorbio Bio-Pharm Technology Co., Ltd. (Shanghai, China), and all data were analyzed on the Meggie BioCloud online platform (www.majorbio.com).

### 2.5. Identification of Chemical Composition

UPLC-Q-TOF-MS/MS was used for identification and analysis. In total, 200 mg of EAEPC was placed in a centrifuge tube with 1 mL of 80% methanol and grinding beads, ground, vortexed, and then centrifuged (20,000× *g*, 10 min, 4 °C). The supernatant was then filtered through a 0.22 μm organic membrane for testing. Measured after setting up according to the following conditions, the data were preliminarily organized with CD 2.1 and compared with database searches (ChemSpider, mzCloud and mzVault) and manual review.

UPLC conditions: Column XB-C18 (50 mm × 2.1 mm, 1.8 μm). Column temperature: 35 °C. Injection volume: 5.0 μL. Mobile phase: A (water, with 0.1% formic acid) and B (methanol). Flow rate: 0.3 mL/min. Elution conditions (A:B): 0.01 min, 98:2; 5 min, 80:20; 10 min, 50:50; 20 min, 5:95; 25 min, 5:95; 26 min, 98:2; and 30 min, the controller stopped. MS conditions: ESI source. Scanning mode: positive and negative switching scanning. Detection mode: full mass/dd-MS2. Resolution: 70,000 (full mass) and 17,500 (dd-MS2). M/Z scanning range: 150.0–2000.0. Electrospray voltage: 3.8 kV (positive). Capillary temperature: 300 °C. Collision gas, sheath gas, and auxiliary gas: Ar and N_2_ (purity ≥ 99.999%), 40 Arb, 350 °C.

### 2.6. Prediction and Validation of Active Ingredient

The potential active substances in EAEPC were predicted through databases such as Traditional Chinese Medicine Systems Pharmacology (TCMSP), the Traditional Chinese Medicine Integrated Database (TCMID), the Encyclopedia of Traditional Chinese Medicine (ETCM), and PubMed, among others. Similarly, disease-associated genes for gastroparesis were retrieved from GeneCards, DisGeNET, Malacard, et al., and the disease gene information was standardized by the Uniprot database. Cross-targeting points were determined using Venn diagrams. Cytoscape software (v3.9.1,) was used to construct and visualize the protein–protein interaction (PPI) network and to calculate and screen the top-10 ingredients with the highest degree of freedom as the potential active ingredients for improving gastroparesis. Subsequently, the predicted major active ingredients were quantified by LC-MS, and their contents and percentages were calculated according to the standard curve equations. Multi-component complex granules (MCCGs) were obtained by compounding, and their effects on gastroparesis mice were compared with those of EAEPC in animal experiments.

The predicted major active ingredients were determined by high-performance liquid chromatography–mass spectrometry 9030 (Shimadzu, Kyoto, Japan). In total, 2 mg of EAEPC was placed in a centrifuge tube with 1 mL of 80% methanol and grinding beads, ground, vortexed, and then centrifuged. The supernatant was then filtered through a 0.22 μm organic membrane for testing. UPLC conditions: Column SHIMADZU C18-AQ (4.6 mm × 150 mm, 5 μm). Column temperature: 40 °C. Injection volume: 5.0 μL. Mobile phase: A (water, with 0.1% formic acid) and B (methanol). Flow rate: 0.5 mL/min. Elution conditions (A:B): 0.01 min, 98:2; 5 min, 80:20; 1 min, 50:50; 7 min, 5:95; 25 min, 5:95; 26 min, 98:2; and 30 min, the controller stopped. The ion source was an ESI, and the parameters were as follows: interface voltage, 4.5 kV; DL temperature, 250 °C; desolvation temperature, 526 °C; interface temperature, 300 °C; heater flow rate, 10.0 L/min; nebulizing gas flow rate, 3.0 L/min; and drying gas flow rate, 10.0 L/min. Afterwards, all processed supernatants were filtered through a 0.22 μm organic membrane before quantification. MRM scanning mode conditions and mass spectral parameters were as shown in Table S4.

### 2.7. Statistical Analysis

SPSS 24.0 was used to statistically analyze the data, and the data were presented as the mean ± standard error of the mean. The data figures and statistical significance analysis (one-way ANOVA was used to evaluate the differences between the groups, taking *p* < 0.05 as the statistically significant difference, and post hoc comparison was implemented through Tukey’s test) were realized with GraphPad Prism software (v9.0 USA).

## 3. Results and Discussion

### 3.1. EAEPC for Gastroparesis Efficacy

**Figure 1:**
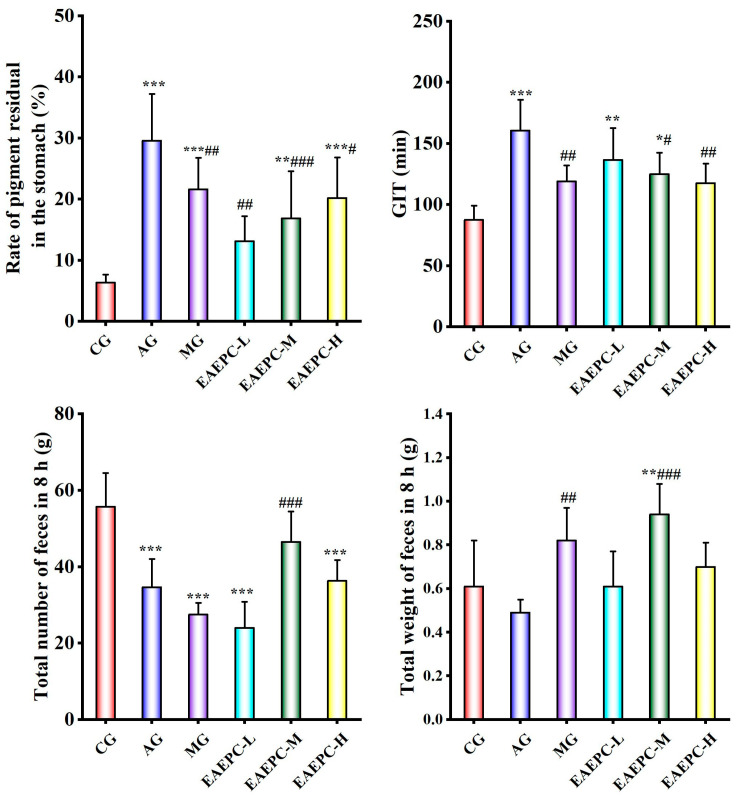
Improvement in gastroparesis with different concentrations of EAEPC (n = 10). Compared to CG: * *p* < 0.05, ** *p* < 0.01, and *** *p* < 0.001. Compared to AG: ^#^ *p* < 0.05, ^##^ *p* < 0.01, and ^###^ *p* < 0.001.

Atropine is a widely used agent for inducing gastroparesis in basic research models. After injecting atropine into the mice, significant differences in the rate of pigment residual in the stomach, GIT, and total number of feces in 8 h were observed in the AG compared to in the CG (*p* < 0.01). Specifically, the rate of pigment residual in the stomach in the AG increased nearly fivefold, the GIT nearly doubled, and the number of defecations decreased by 37.72%, indicating that the gastrointestinal peristalsis of the mice was significantly slowed down after atropine modeling (Figure 1). Notably, compared with the model group, different concentrations of EAEPC improved the levels of various efficacy indexes to different degrees (*p* < 0.05), indicating that EAEPC could improve atropine-induced gastroparesis, and the improvement effect of medium-concentration EAEPC was superior.

The efficacy results indicated that different concentrations of EAEPC could significantly improve various gastrointestinal motility function indexes in mice with gastroparesis to different degrees. Based on this, the coupled coordination model and the weighted RSR model were used to determine the optimal concentration of EAEPC for improving gastroparesis. The combined application of the two mathematical models determined the optimal concentration of EAEPC for improving atropine-induced gastroparesis from multiple perspectives and dimensions, and this mathematical and statistical model can be applied to the comprehensive evaluation of functional foods for multiple efficacy indicators to make it more accurate and credible.

Upon quantifying the data for each efficacy index, the results (Table 1) showed that EAEPC-M had the highest coupled coordination. Additionally, weighted RSR (rank sum ratio) calculations showed that EAEPC-M had higher ranks and grading levels than the others, suggesting superior coordination. In conclusion, the efficacy of different concentrations of EAEPC in improving the symptoms of gastroparesis was ranked as EAEPC-M > EAEPC-H > EAEPC-L by two comprehensive evaluations, indicating that the middle concentration of EAEPC was the most effective in improving gastroparesis induced by atropine. Additionally, the investigation of the mechanism will also be based on the study of EAEPC-M.

### 3.2. Alterations in Major Intestinal Metabolites

**Figure 2:**
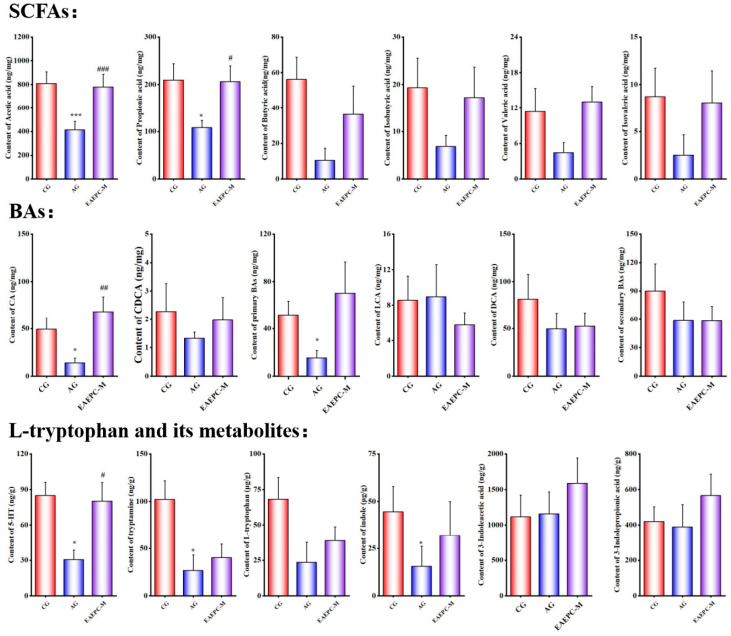
Effects of EAEPC-M on the levels of major metabolites (SCFAs, BAs, and L-tryptophan metabolites) in the feces of gastroparesis mice. Compared to CG: * *p* < 0.05 and *** *p* < 0.001. Compared to AG: ^#^ *p* < 0.05, ^##^ *p* < 0.01, and ^###^ *p* < 0.001.

The alterations in the content of major intestinal metabolites (SCFAs, BAs, L-tryptophan, and its metabolites) in the colonic contents of three groups of mice (CG, AG, and EAEPC-M) are depicted in Figure 2.

#### 3.2.1. Short-Chain Fatty Acids

SCFAs, which are produced by the gut microbiota through the conversion and breakdown of dietary fiber and polysaccharides, have been shown to promote gastrointestinal motility [22]. Compared with the CG, the levels of all six SCFAs in the feces of mice in the AG were reduced to different degrees, among which the levels of acetic acid and propionic acid were significantly reduced (*p* < 0.05), and EAEPC-M restored both individual SCFAs in gastroparesis mice, and there was no significant difference compared with those in the CG. In terms of the total SCFA content, EAEPC-M reached 1058.82 ng/mg, which was almost the same as that of the CG at 1113.03 ng/mg. These results suggest that EAEPC is effective in restoring the levels of individual SCFAs and total SCFAs in the colonic contents of gastroparesis mice to normal range and potentially promotes an increase in the number of genera associated with SCFA production.

#### 3.2.2. Bile Acids

BAs can be classified by structure into free and conjugated BAs. Free BAs include cholic acid (CA), chenodeoxycholic acid (CDCA), deoxycholic acid (DCA), and lithocholic acid (LCA), and if categorized by the source of the BAs, CA and CDCA are primary BAs, while DCA and LCA are secondary BAs. There is a close relationship between BAs and the gut microbiota; primary BAs are metabolized by the gut microbiota in the gastrointestinal tract, resulting in the formation of secondary BAs. These metabolites play a crucial role in promoting gastrointestinal motility by activating bile acid-coupled receptor 5 and stimulating 5-HT secretion [23]. Atropine-induced gastroparesis mice showed an overall decrease in BA levels compared to the CG. However, the content of CA in the colonic contents of gastroparesis mice was significantly increased (*p* < 0.05) after interventions with EAEPC-M. Some studies have demonstrated that an imbalance in the ratio of primary to secondary BAs can also impact gastrointestinal peristalsis [24]. The significant reduction in primary BAs in the AG indicates that the gut microbiota catabolizes and converts primary BAs at a reduced rate, suggesting that the abundance of genera that catabolize primary BAs is increased in the gastroparesis model, which is reversed by EAEPC-M. Therefore, increasing the CA content in the metabolic pathway of BAs and altering the ratio of primary BAs may be one of the mechanisms by which EAEPC improves gastroparesis.

#### 3.2.3. L-Tryptophan and Its Metabolites

L-tryptophan, an essential amino acid obtained from food, has metabolic pathways that mainly include the kynurenine pathway, the 5-HT pathway, and the indole pathway within the gastrointestinal tract [15]. Indole derivatives include indole and indole-3-acetic acid as well as indole-3-propionic acid, which can activate enterochromaffin cells via transient receptor potential fixation protein A1, promote gastrointestinal movement, or act on aryl hydrocarbon receptors to cause an increase in the secretion of 5-HT by enterochromaffin cells. L-tryptophan can also form tryptamine through the action of enzymes; tryptamine increases cyclic AMP release and anion-dependent fluid secretion from proximal colonic epithelial cells [25], and it also stimulates the release of 5-HT from enteric neurons [26].

During the occurrence of gastroparesis, a significant reduction was observed in the levels of L-tryptophan, 5-HT, tryptamine, and indole in the feces of mice (*p* < 0.05), which were about 1/4 to 1/2 of the content of the CG, with little change in the levels of 3-indoleacetic acid and 3-indolepropionic acid. After 7 days of EAEPC-M gavage in gastroparesis mice, the levels of L-tryptophan and its metabolites in the feces of the mice rebounded, especially the level of 5-HT, which was significantly increased (*p* < 0.05) and was not statistically different from that of the CG. 5-HT is a key activator of the gastrointestinal motor reflex by acting on various receptors in the enteric nervous system [27]. The mechanism by which 5-HT participates in gastrointestinal tract function is complex, and it promotes gastrointestinal dynamics mainly through Tph1 and SERTs influencing the 5-HT level and mediating the specific activation of 5-HT_4_ receptors [28]. This result suggests that there is a link between EAEPC-M’s role in promoting 5-HT secretion and improving symptoms in gastroparesis mice.

In summary, in terms of the major metabolites contained in the colonic contents, EAEPC significantly increased the levels of acetic acid, propionic acid, total SCFAs, CA, and 5-HT in the feces of mice with gastroparesis, whereas the levels of secondary BAs and indole and their metabolites did not show significant changes. Therefore, it is possible that EAEPC promotes gastrointestinal motility by regulating the gut microbiota to mediate the secretion of SCFAs, CA, and 5-HT.

### 3.3. Effect on the Gut Microbiota

Numerous studies have shown that the diversity and compositional structure of the gut microbiota are crucial for gastrointestinal dynamics [29]. Therefore, fecal samples from three groups of mice (CG, AG, and EAEPC-M) were also subjected to 16S rRNA high-throughput sequencing to analyze the differences in diversity and composition between different groups of gut microbiota.

#### 3.3.1. Diversity Analysis

**Figure 3:**
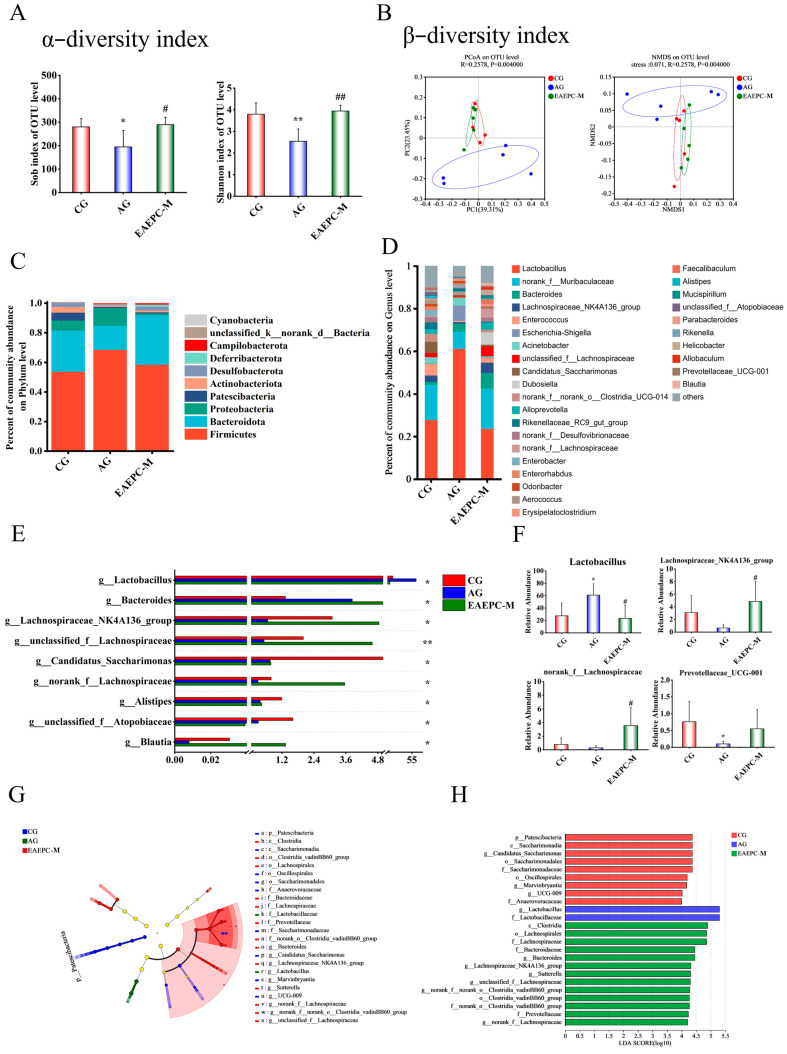
Gut microbiota analysis of mice in each group (CG, AG, and EAEPC-M). (**A**) α-diversity indexes; (**B**) β-diversity indexes; (**C**) relative abundance at the phylum level; (**D**) relative abundance at the genus level; (**E**) LEfSe taxonomic cladograms; (**F**) LDA distribution of different species; (**G**) LefSe taxonomic cladogram; (**H**) LDA distribution of different species. Compared to CG: * *p* < 0.05, and ** *p* < 0.01. Compared to AG: ^#^ *p* < 0.05, and ^##^ *p* < 0.01.

The results of α-diversity and β-diversity analyses are shown in Figure 3A,B. The significant decrease in α-diversity indexes (Sob and Shannon) between the AG and CG mice (*p* < 0.05) indicates a lower richness and species diversity of the gut microbiota in gastroparesis mice. Conversely, the Sob, Shannon, and Chao diversity indices of the EAEPC-M intervention group were significantly higher (*p* < 0.05) than those of the AG, suggesting that gavage with EAEPC-M was able to restore the diversity of the gut microbiota species in gastroparesis mice. According to Figure 3B, the Principal Co-ordinate Analysis (PCoA) plot shows the overall differences in the gut microbiota of mice among different groups. As can be seen from the graph, the CG and AG were farther apart, indicating that atropine-induced gastroparesis caused significant changes in the structure of the gut microbiota in mice. After the administration of EAEPC, there was an overlap between the samples from the EAEPC-M group and CG, indicating that EAEPC-M could regulate the species composition structure of the intestinal microbiota of the gastroparesis mice, making them closer to the CG. The Non-metric Multidimensional Scaling (NMDS) clustering plot was used to show differences in the microbial composition among the three groups; the stress value for this model was 0.071 (a stress value below 0.1 in NMDS calculations indicates that the model is of good quality and the classification is reliable). In Figure 3, while there is a small overlap between the AG and CG, samples from the EAEPC-M group showed a larger degree of overlap with those from the CG; this suggests that EAEPC-M has the potential to ameliorate gastroparesis by remodeling the gut microbiota structure, as observed by PCoA.

#### 3.3.2. Species Composition and Differential Analysis

*Firmicutes* and *Bacteroidota* are often used as some of the most important predictors of gastrointestinal disease [30], with *Firmicutes* playing an important role in host and intestinal homeostasis, normalizing intestinal permeability, and being involved in the regulation of the cerebral–gut axis, while *Bacteroidota* contributes to the catabolism of dietary fiber and starch to release energy and in some cases participates in the metabolism of BAs. As shown in Figure 3C, at the phylum level, compared to the CG, the relative abundance of *Proteobacteria* increased, while *Bacteroidota* decreased in the feces of gastroparesis mice; however, both were regressed to levels similar to those seen in the CG after EAEPC-M intervention. An increase in the number of Proteobacteria may affect the normal functioning of nerves and muscles within the gastrointestinal tract, leading to slowed or irregular gastrointestinal motility. This may result in symptoms such as constipation and diarrhea [31]. The results suggest that EAEPC-M intervention resulted in a healthier gut microbiota in mice.

To further understand the changes in the relative abundance of the gut microbiota in each group of mice at the genus level, we also analyzed their composition, as shown in Figure 3D. After calculation, it was discovered that nine taxa, including *Lactobacillus*, *Bacteroides*, and *Lachnospiraceae_NK4A136_group*, differed considerably across the CG, AG, and EAEPC-M group (Figure 3E). As shown in Figure 3F, among the substantially modified strains, the relative abundance of *Lactobacillus* increased significantly to 61.03% in the AG but fell to 23.53% following EAEPC-M intervention (*p* < 0.05), which was equivalent to that of the CG. In contrast, the relative abundances of *Lachnospiraceae_NK4A136_group*, *norank_f__Lachnospiraceae,* and *Prevotellaceae_UCG-001* decreased in gastroparesis mice but increased after EAEPC-M intervention (*p* < 0.05), with levels not varying statistically from the CG. As shown in Figure 3G and 3H, LEfSe analysis was conducted based on LDA scores greater than 4, revealing that 24 identified gut microbiota from the phylum to genus levels had a greater impact on the microbial community structure. Three groups respectively screened out 7, 2, and 13 significantly different bacteria. These results indicate that after EAEPC intervention, compared with the gut microbiota of mice in the CG and AG groups, the marker bacterial species were more abundant.

### 3.4. The Correlation and Mechanism of Major Intestinal Metabolites and the Gut Microbiota

**Figure 4:**
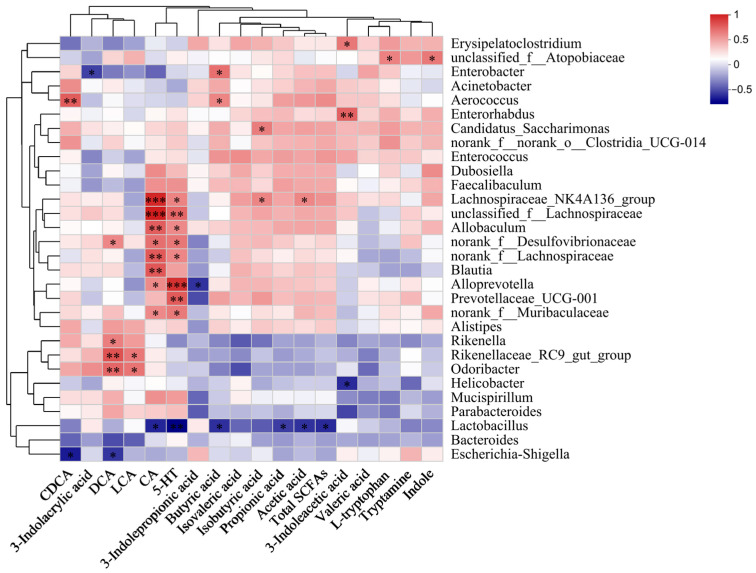
Correlation analysis between gut microbiota and its metabolites in mice with gastroparesis after EAEPC intervention (* *p* < 0.05, ** *p* < 0.01, *** *p* < 0.001).

Spearman correlation analysis was used to elucidate the interplay between the gut microbiota and its metabolites in gastroparesis mice after EAEPC intervention (Figure 4). Notably, 5-HT levels showed a positive correlation (*p* < 0.01) with *Lachnospiraceae_NK4A136_group*, *Alloprevotella, and norank_f_Lachnospiraceae*, while CA levels mirrored this trend with *Allobaculum, Lachnospiraceae_NK4A136_group*, and *unclassified_f_Lachnospiraceae* (*p* < 0.01). Acetic acid correlated positively with *Lactobacillus* and inversely with *norank_f_Lachnospiraceae* (*p* < 0.05). From the perspective of the gut microbiota, *Lactobacillus* had a negative correlation with acetic acid, propionic acid, total SCFAs, CA, and 5-HT in fecal samples (*p* < 0.05), whereas *Alloprevotella* showed an inverse relationship with 3-indolepropionic acid and a positive one with CA and 5-HT (*p* < 0.05). Furthermore, *Odoribacter* was positively associated with DCA and LCA levels. These correlations underscore the role of the gut microbiota in metabolite regulation following EAEPC intervention, which may eventually promote gastrointestinal motility and health benefits in atropine-induced gastroparesis mice.

Functional foods, known for their gentle, healthy properties, have shown promising results in promoting gastrointestinal motility, and their use as complementary and alternative therapies has gained increasing attention. It is well known that gastrointestinal motility is a complex and critical process involving the co-regulation of the gastrointestinal nervous system, endocrine system, and microbiota. The gut microbiota plays a key role in maintaining gastrointestinal motility, and its relationship with major intestinal metabolites is particularly strong, especially with SCFAs, BAs, and L-tryptophan metabolites.

**Figure 5:**
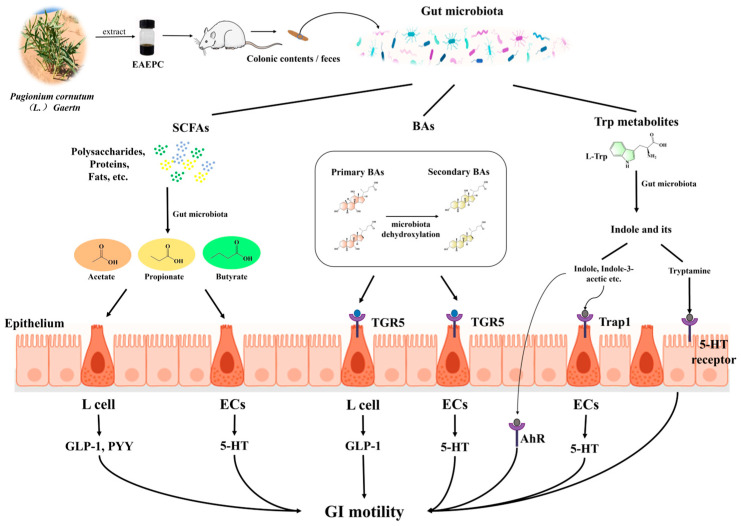
Modulatory mechanism of EAEPC improving gastroparesis via gut microbiota and its metabolites.

The regulatory mechanism of EAEPC to improve gastroparesis by regulating metabolites through the gut microbiota may be as follows (Figure 5): EAEPC helps regulate the relative abundance of *Firmicutes* and *Bacteroidetes* (phylum level), *Lactobacillus*, *Lachnospiraceae_NK4A136_group*, *norank_f__Lachnospiraceae* (genus level), and *Alloprevotella*, which increases ① the content of acetic acid, propionic acid, and butyric acid, which can enhance gastrointestinal motility by upregulating tryptophan hydroxylase 1 (Tph1) expression and increase 5-HT secretion [32]. At the same time, an increase in total SCFAs promotes plasma glucagon-like peptide-1 (GLP-1) secretion, significantly promoting gastric emptying and intestinal propulsion [33]. ② The content of CA thereby further stimulates 5-HT secretion and accelerates gastrointestinal peristaltic movement along with gastric emptying in gastroparesis mice, and this process was associated with increased Tph1 expression [34]. ③ The content of 5-HT suggests that EAEPC may enhance the expression of Tph1 because Tph1 is the key rate-limiting enzyme for 5-HT synthesis [35]. Importantly, this entire process is likely mediated by the gut microbiota without involving the indole metabolic pathway. However, this requires further investigation.

### 3.5. Identification, Prediction, and Verification of Main Active Ingredients

EAEPC components were identified through UPLC-Q-TOF-MS/MS (Figure S1). A total of 102 compounds were identified in positive-ion mode (Table S2-1), including adenine, betaine, esculetin, and others, while 32 compounds were identified in negative-ion mode, including caffeic acid, rutin, astragalin, quercetin, and others (Table S2-2). A total of 119 compounds were obtained after weight removal, dominated by flavonoids and organic acids.

**Figure 6:**
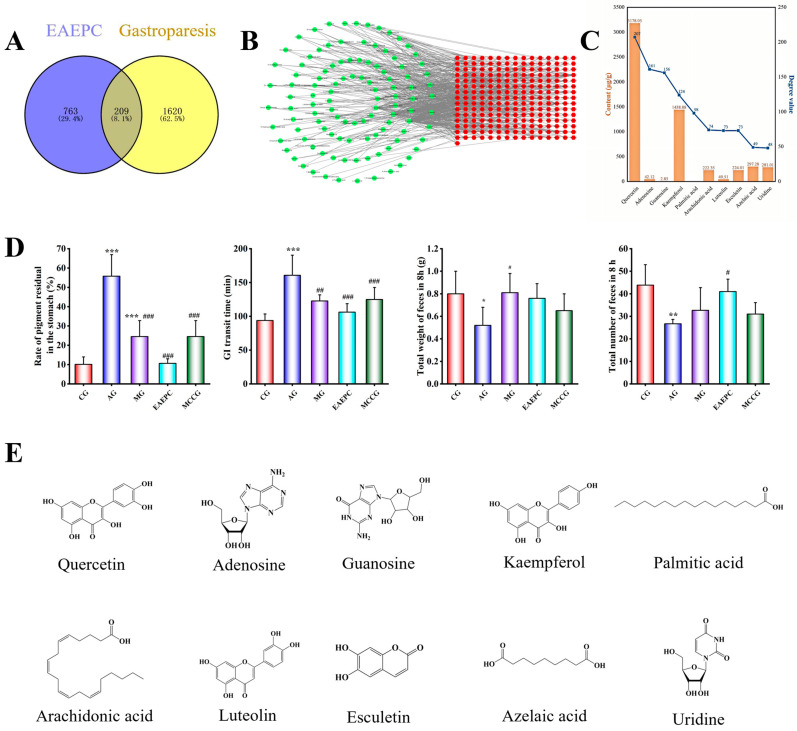
(**A**) Venn diagram of core targets for EAEPC to improve gastroparesis; (**B**) PPI network of key targets; (**C**) number and content of targets for the top-10 substances according to the degree value; (**D**) improvement in MCCGs in gastroparesis mice; (**E**) chemical structural formulas of 10 potentially active substances. Compared to CG: * *p* < 0.05, ** *p* < 0.01, and *** *p* < 0.001. Compared to AG: ^#^ *p* < 0.05, ^##^ *p* < 0.01, and ^###^*p* < 0.001.

After filtering the findings using the TCMSP, TCMID, ECTM, TargetNet, and PubMed databases, 112 compounds were found, along with 972 associated therapeutic targets. Additionally, 1829 gastroparesis-related disease targets were gathered from the Disgenet, Genecard, and Malacard databases. The therapeutic targets for compounds of EAEPC were mapped to gastroparesis disease targets, yielding 226 overlapping targets, as shown in the Venn diagram in Figure 6A. These intersecting targets were further analyzed in the String database to establish the PPI network (Figure 6B). Based on the topological parameters of the PPI network (Table S3), 10 compounds (Figure 6E) with high degrees of freedom values were obtained as potentially active substances and quantitatively analyzed using LC-MS (Figure 6C). The quantitative results revealed that the contents of quercetin and kaempferol were significantly higher than those of the other components, followed by azelaic acid and arachidonic acid; however, no palmitic acid was identified; quercetin can promote gastrointestinal motility and mucin secretion by modulating muscarinic acetylcholine receptor signaling pathways [36], while kaempferol enhances signaling in the intestinal epithelium, reducing GIT in mice [37]; luteolin has been found to stimulate the contraction of isolated smooth muscle strips in the gastrointestinal tract [38]; arachidonic acid is converted to prostaglandins by cyclooxygenase catalysis, which stimulates strong contraction of the longitudinal muscles of the colon and promotes gastrointestinal motility [39].

The mixture (MCCGs) was obtained by determining and calculating the proportion of each potentially active substance in the EAEPC, using animal experiments to validate the efficacy of the action. In gastroparesis mice, there was no significant difference in the organ indices between the AG, MG, EAEPC, MCCGs, and CG, showing that EAEPC and MCCGs had no harmful effects on the organs and tissues of gastroparesis mice. As demonstrated in Figure 6D, following the intervention, the rate of pigment residue in the stomach and GIT was greatly reduced; the total number and weight of feces in 8 h were dramatically increased, indicating a significant difference from the AG (*p* < 0.05). MCCGs improved these four indices in gastroparesis mice by 87.40%, 65.45%, 30.22%, and 54.42%, respectively, compared to EAEPC. MCCGs composed of nine compounds may considerably increase gastric motility with a total effectiveness of 59.37% compared to EAEPC; this shows that these nine compounds (quercetin, adenosine, guanosine, kaempferol, arachidonic acid, esculetin, luteolin, azelaic acid, and uridine) from *Pugionium* are the main active ingredients that improve gastroparesis.

## 4. Conclusions

This study, for the first time, confirms that the extract of *Pugionium*, a traditional Mongolian vegetable, has demonstrated efficacy in alleviating gastroparesis. EAEPC may help with gastroparesis by modulating the structure and diversity of the gut microbiota and moderating the levels of intestinal metabolites (SCFAs, CA, and 5-HT). Furthermore, quercetin, kaempferol, adenosine, and arachidonic acid were also identified as the main pharmacodynamic components of *Pugionium* to improve gastroparesis. This may be the main reason why EAEPC improves gastroparesis and promotes gastrointestinal motility by mediating metabolites through the gut microbiota. This study presents a comprehensive and reliable chain of evidence, demonstrating EAEPC’s efficacy as a plant-derived extract for gastroparesis and proposing a new idea for alternative treatments for gastroparesis.

In summary, this study determined that EAEPC regulates the secretion of SCFAs, CA, and 5-HT by regulating the composition of the gut microbiota and improving the symptoms of gastroparesis. However, further studies are needed to fully elucidate the complex regulatory mechanisms involved and evaluate the potential application of *Pugionium* extract in improving gastrointestinal motility disorders.

## Figures and Tables

**Table 1:** Calculation results of coupling coordination degree method and weighted RSR method.

**Coupling Coordination Degree Method**				
Group	Coupling C-value	Harmonization Index T-value	Coupling Coordination Degree D-value	Coordination level
EAEPC-L	0.193	0.448	0.294	3
EAEPC-M	0.462	0.536	0.498	5
EAEPC-H	0.245	0.425	0.323	4
**Weighted RSR Method**				
Group	RSR value	RSR rank	RSR Fitting value	Staging Level
EAEPC-L	0.667	2	0.665	2
EAEPC-M	0.704	1	0.704	3
EAEPC-H	0.630	3	0.630	2

## Data Availability

All relevant data are within the manuscript and its Supplementary Materials.

## Supplementary Materials

The following supporting information can be downloaded at https://www.mdpi.com/article/10.3390/foods14162800/s1: Table S1: MRM conditions and mass spectral parameters for major metabolites (SCFAs, BAs, and L-tryptophan and its metabolites). Figure S1: Negative- and positive-mode total flow diagram based on UPLC-Q-TOF-MS/MS identification and analysis of EAEPC components. Table S2-1: Components identified from EAEPC by UPLC-Q-TOF-MS/MS under negative-ion mode. Table S2-2: Components identified from EAEPC by UPLC-Q-TOF-MS/MS under positive-ion mode. Table S3: Topological freedom of compounds in EAEPC based on PPI networks. Table S4: MRM conditions and mass spectral parameters for major active ingredients.

## Abbreviations

The following abbreviations are used in this manuscript:

EAEPC	Extract from *Pugionium cornutum* (L.) Gaertn
SCFAs	Short-chain fatty acids
BAs	Bile acids
EPC	Ethanolic extract of *Pugionium*
CG	Control group
AG	Model group (atropine)
EAEPC-H/M/L	EAEPC high, medium, and low concentration
GIT	Gastrointestinal transit time
MRM	Multi-reaction monitoring
TCMSP	Traditional Chinese Medicine Systems Pharmacology
TCMID	Traditional Chinese Medicine Integrated Database
ETCM	The Encyclopedia of Traditional Chinese Medicine
PPI	Protein–protein interaction
MCCGs	Multi-component complex granules
CA	Cholic acid
CDCA	Chenodeoxycholic acid
DCA	Deoxycholic acid
LCA	Lithocholic acid
5-HT	5-hydroxytryptamine
PCoA	Principal Co-ordinate Analysis
NMDS	Non-metric Multidimensional Scaling
