# Supplementary material for: Functions of Pugionium cornutum (L.) Gaertn Extracts: Investigating the Mechanism of Gastroparesis Amelioration from the Perspective of the Gut Microbiota and Its Metabolites
Source: Foods. 2025 Aug 12;14(16):2800. doi: 10.3390/foods14162800 (PMC12386120; doi:10.3390/foods14162800)
Supplement: Supplementary file 1 [file foods-14-02800-s001.zip › foods-3753823-supplementary.pdf]

Supplementary information

Table S1 MRM conditions and mass spectral parameters for major metabolites 2
Figure S1 Negative and positive mode total flow diagram based on UPLC-Q-TOF-MS/MS identification and analysis of EAEPC components 3
Table S2-1 Components identified from EAEPC by UPLC-Q-TOF-MS/MS under positive ion mode...4
Table S2-2 Components identified from EAEPC by UPLC-Q-TOF-MS/MS under negative ion mode. 12
Table S3 Topological freedom of compounds in EAEPC based on PPI networks 14
Table S4 MRM conditions and mass spectral parameters for major active ingredients......17

Table S1 MRM conditions and mass spectral parameters for major metabolites

Class	Name	Precursor ion (m/z)	Product ion (m/z)	CE（ev）	t_R (min)
SCFAs	Acetic acid	194.0600	137.0360	18	4.933
	Propionic acid	208.0700	137.0360	17	6.890
	Isobutyric acid	222.0900	137.0360	17	15.547
	Butyric acid	222.0900	137.0360	17	10.480
	Isovaleric acid	236.1000	137.0360	20	18.500
	Valeric acid	236.1000	137.0360	20	16.822
Free BAs	Cholic acid	407.2843	-	25	15.787
	Chenodeoxycholic acid	391.2895	-	25	19.332
	Deoxycholic acid	391.2891	-	25	19.815
	Lithocholic acid	375.2956	-	25	25.428
L-tryptophan and its metabolites	5-HT	177.1024	160.0760	6	9.855
	Tryptamine	161.1069	144.0811	6	11.655
	L-Tryptophan	205.0977	188.0711	8	11.412
	Indole	118.0651	91.0534	24	11.750
	3-Indoleacetic acid	176.0709	130.0652	11	17.560
	3-Indolepropionic acid	190.0867	130.0654	11	19.467
	3-Indoleacrylic acid	188.0706	170.0604	8	18.203

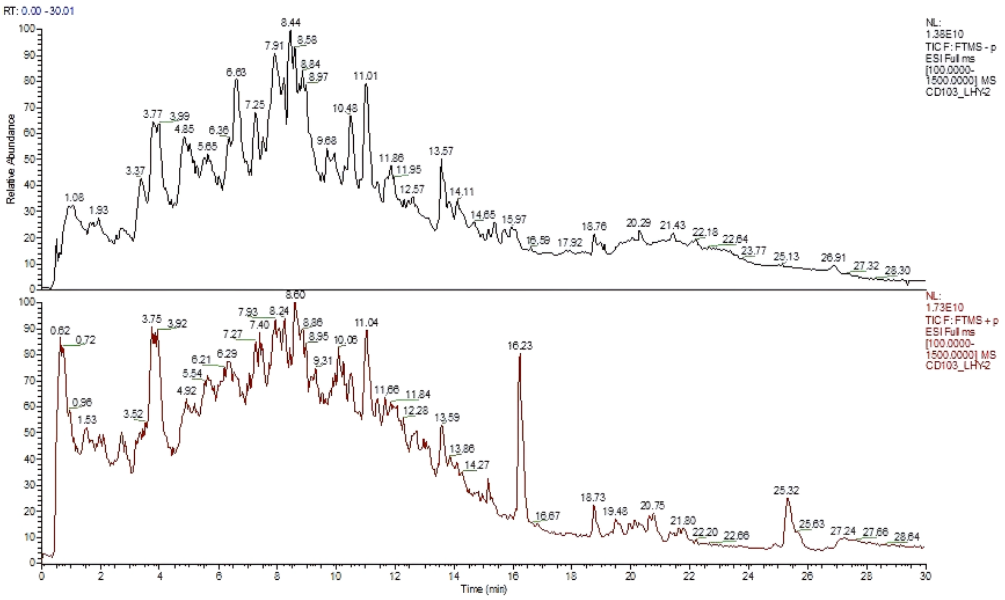

Figure S1 Negative and positive mode total flow diagram based on UPLC-Q-TOF-MS/MS identification and analysis of EAEPC components

Table S1-1 Components identified from EAEPC by UPLC-Q-TOF-MS/MS under positive ion mode.

No.	t_R (min)	Identification	Elemental compoisition	Proposed Ions	Experimental Mass (m/z)	Theoretical Mass (m/z)	Mass Error (ppm)	**MS/MS(m/z)**
1	0.43	D-Glucosamine	$C_6H_{13}NO_5$	$[M+H]^+$	180.0864	179.0794	-1.6659	70.06586, 116.07075, 88.03986, 162.07584
2	0.45	Choline	$C_5H_{13}NO$	$[M+H]^+$	104.1072	103.0997	1.9211	60.08156, 58.06594, 87.04454
3	0.53	L-Histidine	$C_6H_9N_3O_2$	$[M+H]^+$	156.0766	155.0695	-1.2814	83.06094, 95.06079, 110.07145
4	0.54	3-Aminophenol	C_6H_7NO	$[M+H]^+$	110.0602	109.0529	0.0000	82.06566, 92.04985
5	0.56	Adenine	$C_5H_5N_5$	$[M+H]^+$	136.0615	135.0545	-2.2049	119.02653, 94.04027
6	0.56	Cytosine	$C_4H_5N_3O$	$[M+H]^+$	112.0508	111.0433	1.7849	95.02431, 69.04540
7	0.57	DL-Arginine	$C_6H_{14}N_4O_2$	$[M+H]^+$	175.1188	174.1115	0.0000	70.06585, 60.05647, 116.07074, 130.09738
8	0.58	DL-Stachydrine	$C_7H_{13}NO_2$	$[M+H]^+$	144.1017	143.0946	-1.3879	70.06580, 84.04480, 58.06594
9	0.60	2'-Deoxyadenosine	$C_{10}H_{13}N_5O_3$	$[M+H]^+$	252.1086	251.1018	-1.9833	136.06157, 117.05471
10	0.62	Pyridoxine	$C_8H_{11}NO_3$	$[M+H]^+$	170.0809	169.0739	-1.7639	152.07028, 134.05984, 124.07565
11	0.66	Guanine	$C_5H_5N_5O$	$[M+H]^+$	152.0565	151.0494	-1.3153	110.03511, 135.03000, 81.00113
12	0.68	Proline	$C_5H_9NO_2$	$[M+H]^+$	116.0707	115.0633	0.8615	70.06583
13	0.73	Nicotinic acid	$C_6H_5NO_2$	$[M+H]^+$	124.0393	123.0320	0.0000	80.05006, 96.04478
14	0.74	Betaine	$C_5H_{11}NO_2$	$[M+H]^+$	118.0864	117.0790	0.8468	72.08144, 59.07377, 55.01871
15	0.84	1-Methylguanine	$C_6H_7N_5O$	$[M+H]^+$	166.0723	165.0651	-0.6021	149.04550, 153.04050, 124.05037

16	0.85	Valylproline	$C_{10}H_{18}N_2O_3$	$[M+H]^+$	215.1388	214.1317	-0.9296	70.06583, 72.08147, 137.07072, 169.13339
17	0.93	L-Norleucine	$C_6H_{13}NO_2$	$[M+H]^+$	132.1018	131.0946	-0.7570	86.09694, 69.07062
18	0.97	L-Pyroglutamic acid	$C_5H_7NO_3$	$[M+H]^+$	130.0499	129.0426	0.0000	84.04491, 56.05033
19	1.37	6-Quinolinecarboxylic acid	$C_{10}H_7NO_2$	$[M+H]^+$	174.0548	173.0477	-1.1491	146.05974, 130.06497
20	1.40	Thymine	$C_5 H_6N_2O_2$	$[M+H]^+$	127.0502	126.0429	0.0000	110.02380, 109.03983, 84.04490
21	1.53	Phenethylamine	$C_8H_{11}N$	$[M+H]^+$	122.0965	121.0892	0.0000	105.07012, 103.05450, 79.05484
22	1.63	Adenosine	$C_{10}H_{13}N_5O_4$	$[M+H]^+$	268.1035	267.0962	0.0000	136.06158, 57.03439
23	1.64	Quinoline	C_9H_7N	$[M+H]^+$	130.0652	129.0579	0.0000	84.04494, 103.05453, 56.05033
24	1.91	L-Phenylalanine	$C_9H_{11}NO_2$	$[M+H]^+$	166.0860	165.079	-1.8063	77.03940, 103.05450, 120.08080
25	2.04	4-Pyridoxic acid	$C_8H_9NO_4$	$[M+H]^+$	184.0603	183.0532	-1.0866	166.04953, 148.03899, 184.06026
26	2.20	Guanosine	$C_{10}H_{13}N_5O_5$	$[M+H]^+$	284.0985	283.0917	-1.7599	150.04085, 133.01424, 282.08365
27	2.41	4-Aminobenzoic acid	$C_7H_7NO_2$	$[M+H]^+$	138.0549	137.0477	-0.7243	121.02846, 95.04955, 94.06554
28	2.74	Anthranilic acid	$C_7H_7NO_2$	$[M+H]^+$	138.0548	137.0477	-1.4487	94.06550, 120.04440, 95.04951
29	3.17	2'-O-Methyladenosine	$C_{11}H_{15}N_5O_4$	$[M+H]^+$	282.1193	281.1124	-1.4178	136.06157, 101.03532, 69.03423
30	3.64	cis,cis-Muconic acid	$C_6H_6O_4$	$[M+H]^+$	143.0339	142.0266	0.0000	69.07057, 97.06520, 125.02329
31	3.73	6-Methylquinoline	$C_{10}H_9N$	$[M+H]^+$	144.0806	143.0735	-1.3881	117.06998, 98.06040, 70.06582

32	3.84	2,4-Quinolinediol	$C_9H_7NO_2$	$[M+H]^+$	162.0549	161.0477	-0.6171	116.04956, 134.05989, 144.05414
33	3.89	Trigonelline	$C_7H_7NO_2$	$[M+H]^+$	138.0549	137.0477	-0.7243	67.05491, 95.04946, 65.03929
34	4.03	Indole-3-acrylic acid	$C_{11}H_9NO_2$	$[M+H]^+$	188.0734	187.0631	15.9515	146.05975, 118.06519, 188.07036
35	4.03	1,5-Naphthalenediamine	$C_{10}H_{10}N_2$	$[M+H]^+$	159.0916	158.0844	-0.6286	132.08069, 130.06509, 117.05745
36	4.05	4-Indolecarbaldehyde	C_9H_7NO	$[M+H]^+$	146.0599	145.0526	0.0000	118.06522, 146.05984, 91.05471
37	4.07	Caprolactam	$C_6H_{11}NO$	$[M+H]^+$	114.0916	113.0841	1.7530	72.08144, 55.05509, 69.07059
38	4.52	N-Acetyldopamine	$C_{10}H_{13}NO_3$	$[M+H]^+$	196.0968	195.0895	0.0000	137.05951, 91.05467, 119.04922
39	4.72	8-Hydroxyquinoline	C_9H_7NO	$[M+H]^+$	146.0600	145.0528	-0.6846	118.06521, 104.04974
40	4.76	1,5-Isoquinolinediol	$C_9H_7NO_2$	$[M+H]^+$	162.0549	161.0477	-0.6171	116.04954, 144.04416, 134.05984
41	4.93	Salicylic acid	$C_7H_6O_3$	$[M+H]^+$	139.0389	138.0317	-0.7192	95.04950, 121.02841, 67.05494, 105.04501
42	5.86	Sinapine	$C_{16}H_{23}NO_5$	$[M+H]^+$	310.1643	309.1576	-1.9345	251.09081, 175.03874, 147.04384
43	6.03	Acetophenone	C_8H_8O	$[M+H]^+$	121.0650	120.0575	1.6520	93.07027, 103.05449, 91.05466
44	6.42	Esculetin	$C_9H_6O_4$	$[M+H]^+$	179.0338	178.0266	-0.5586	123.04407, 133.02831, 151.03871
45	6.64	Xanthurenic acid	$C_{10}H_7NO_4$	$[M+H]^+$	206.0446	205.0375	-0.9707	178.04958, 132.04428, 188.14305

46	6.85	BMK methyl glycidate	$C_{11}H_{12}O_3$	$[M+H]^+$	193.0859	192.0786	0.0000	161.05939, 105.07011, 133.06464
47	6.99	trans-Cinnamaldehyde	C_9H_8O	$[M+H]^+$	133.0648	132.0575	0.0000	105.07006, 79.05480
48	7.10	3-Methoxybenzaldehyde	$C_8H_8O_2$	$[M+H]^+$	137.0597	136.0524	0.0000	109.06499, 81.07044, 91.05466
49	7.14	Syringic acid	$C_9H_{10}O_5$	$[M+H]^+$	199.0600	198.0528	-0.5024	140.04648, 95.04950, 155.06999
50	7.22	N-Acetyl-L-leucine	$C_8H_{15}NO_3$	$[M+H]^+$	174.1124	173.1052	-0.5743	86.09690, 128.10690, 69.07058
51	7.28	5-Hydroxyindole-3-acetic acid	$C_{10}H_9NO_3$	$[M+H]^+$	192.0654	191.0582	-0.5207	146.05969, 174.05470, 128.04933
52	7.50	N-Acetyltyramine	$C_{10}H_{13}NO_2$	$[M+H]^+$	180.1018	179.0946	-0.5552	121.06477, 138.09106, 94.06551
53	7.57	Isoleucine	$C_6H_{13}NO_2$	$[M+H]^+$	132.1020	131.0946	0.7570	86.09694, 69.07062, 105.07014
54	7.93	Sinapinic acid	$C_{11}H_{12}O_5$	$[M+H]^+$	225.0754	224.0685	-1.7772	207.06480, 175.03873, 119.04920
55	7.94	2-Hydroxycinnamic acid	$C_9H_8O_3$	$[M+H]^+$	165.0546	164.0473	0.0000	147.04375, 119.04918, 91.05466
56	7.99	Coumarin	$C_9H_6O_2$	$[M+H]^+$	147.0440	146.0368	-0.6801	119.04920, 91.05468, 65.03934
57	8.01	5-Ethylcyclohexane-1,3-dione	$C_8H_{12}O_2$	$[M+H]^+$	141.0909	140.0837	-0.7088	95.08588, 67.05495, 123.08044
58	8.12	N-Acetyl-DL-tryptophan	$C_{13}H_{14}N_2O_3$	$[M+H]^+$	247.1074	246.1004	-1.2140	144.04414, 159.09137, 188.07028
59	8.31	Quercitrin	$C_{21}H_{20}O_{11}$	$[M+H]^+$	449.1070	448.1006	-2.0040	303.04922, 229.04912, 137.02322
60	8.65	Indole	C_8H_7N	$[M+H]^+$	118.0654	117.0581	0.0000	91.05461, 72.08143

61	8.67	Ferulic acid	$C_{10}H_{10}O_4$	$[M+H]^+$	195.0651	194.0579	-0.5126	89.03905, 117.03358,135.04395
62	8.68	Afzelin	$C_{21}H_{20}O_{10}$	$[M+H]^+$	433.1122	432.1057	-1.8471	287.05438, 153.01801, 121.02847
63	8.69	Desthiobiotin	$C_{10}H_{18}N_2O_3$	$[M+H]^+$	215.1388	214.1315	0.0000	197.12814, 179.11765
64	8.90	4-Phenylbutyric acid	$C_{10}H_{12}O_2$	$[M+H]^+$	165.091	164.0837	0.0000	147.04372, 119.04914, 91.05464
65	8.92	Quercetin	$C_{15}H_{10}O_7$	$[M+H]^+$	303.0494	302.0427	-1.9799	153.01801, 137.02325, 229.04915
66	8.93	Matairesinol	$C_{20}H_{22}O_6$	$[M+H]^+$	359.1485	358.1416	-1.1137	131.04901, 103.05447, 163.07501
67	9.45	Rutin	$C_{27}H_{30}O_{16}$	$[M+H]^+$	611.1586	610.1534	-3.4361	303.04916, 287.05441, 85.0891
68	9.98	Methenolone	$C_{20}H_{30}O_2$	$[M+H]^+$	303.2314	302.2246	-1.6489	81.07047, 105.07017, 93.07031
69	9.99	(9cis)-Retinal	$C_{20}H_{28}O$	$[M+H]^+$	285.2210	284.2140	-1.0518	199.14780, 157.10088, 81.07047
70	10.03	3-Methyl-5-[(1S,2R,4aR)-1,2,4a,5-tetramethyl-7-oxo-1,2,3,4,4a,7,8,8a-octahydro-1-naphthalenyl]pentanoic acid	$C_{20}H_{32}O_3$	$[M+H]^+$	321.2418	320.2351	-1.8677	81.07049, 151.11153, 107.08582, 93.07032
71	10.07	Kaempferol-3-O-β-glucopyranosyl-7-O-α-rhamnopyranoside	$C_{27}H_{30}O_{15}$	$[M+H]^+$	595.1634	594.1585	-4.0325	287.05432, 85.02894
72	10.51	(+/-)-C75	$C_{14}H_{22}O_4$	$[M+H]^+$	255.1588	254.1518	-1.1757	219.13734, 79.05483, 105.07016
73	10.78	Kaempferol	$C_{15}H_{10}O_6$	$[M+H]^+$	287.0536	286.0477	-4.8771	153.01805, 121.02850

74	10.82	Isophorone	$C_9H_{14}O$	$[M+H]^+$	139.1117	138.1045	-0.7188	69.07056, 93.07025, 95.08588
75	10.92	Ambrosic acid	$C_{15}H_{20}O_4$	$[M+H-H_2O]^+$	264.1362	247.1326	5.3003	187.11143, 201.12695, 229.12178
76	11.38	(3S)-3-Methyl-5-[(1S,8aR)-2,5,5,8a-tetramethyl-4-oxo-1,4,4a,5,6,7,8,8a-octahydro-1-naphthalenyl]pentanoic acid	$C_{20}H_{32}O_3$	$[M+H]^+$	321.2418	320.2351	-1.8677	81.07044, 93.07029, 107.08574, 151.11147
77	11.85	19-Norandrostenedione	$C_{18}H_{24}O_2$	$[M+H]^+$	273.1846	272.1776	-1.0982	145.10098, 131.08537, 105.07021
78	12.65	13,14-Dihydro prostaglandin E1	$C_{20}H_{36}O_5$	$[M+H-H_2O]^+$	356.2563	339.2524	4.7719	81.07049, 151.11156, 107.08578
79	12.69	10-HAD	$C_{10}H_{18}O_3$	$[M+H-H_2O]^+$	186.1256	169.1221	6.9846	81.07044, 123.11681, 109.10139
80	12.75	Mesterolone	$C_{20}H_{32}O_2$	[M+H]+	305.2469	304.2393	0.9828	81.07045, 93.07030, 107.08574
81	13.60	12-Oxo phytodienoic acid	$C_{18}H_{28}O_3$	$[M+H]^+$	293.2103	292.203	0.0000	275.20001, 81.07043, 67.05495
82	13.92	Corchorifatty acid F	$C_{18}H_{32}O_5$	$[M+H]^+$	328.225	327.2172	1.5233	183.13811, 85.02795, 211.13316
83	13.97	OPEO	$C_{16}H_{26}O_2$	$[M+H]^+$	251.2000	250.1933	-2.3885	95.08589, 81.07043, 107.08573
84	14.00	21-Amino-3,20-dihydroxy-2-docosanyl hexopyranoside	$C_{28}H_{57}NO_8$	$[M+H]^+$	536.4146	535.4084	-2.0506	374.36191, 338.34082, 356.35126
85	15.15	Arachidonic acid	$C_{20}H_{32}O_2$	$[M+H-H_2O]^+$	304.2402	287.2362	5.9164	201.16354, 119.08566, 287.23645
86	15.54	Ageratriol	$C_{15}H_{24}O_3$	$[M+H-H_2O]^+$	252.1725	235.1687	6.3449	57.07074, 179.10638

87	15.77	Tetranor-12R-HETE	$C_{16}H_{26}O_3$	$[M+H-H_2O]^+$	266.1882	249.1843	6.3865	193.12210, 137.05949, 105.07009
88	15.95	Bis(4-ethylbenzylidene)sorbitol	$C_{24}H_{30}O_6$	$[M+H]^+$	415.2104	414.2026	1.2042	119.08560, 91.05469, 133.06491
89	16.24	Dibutyl phthalate	$C_{16}H_{22}O_4$	$[M+H]^+$	279.1579	278.1518	-4.2986	149.02312, 65.03931, 121.02839
90	16.44	Citroflex 4	$C_{18}H_{32}O_7$	$[M+H]^+$	361.2206	360.2134	-0.2768	68.99781, 129.01811, 185.08057
91	17.07	Citroflex A-4	$C_{20}H_{34}O_8$	$[M+H]^+$	403.2314	402.2254	-3.2239	129.01810, 185.08054, 147.0285
92	18.59	Linoleoyl ethanolamide	$C_{20}H_{37}NO_2$	$[M+H]^+$	324.2888	323.2824	-2.7753	62.06085, 81.07046, 95.08591
93	19.19	Hexadecanamide	$C_{16}H_{33}NO$	$[M+H]^+$	256.2627	255.2554	0.0000	88.07617, 57.07071, 102.09163
94	19.47	Avocadyne 1-acetate	$C_{19}H_{34}O_4$	$[M+H-H_2O]^+$	326.2457	309.2416	5.8239	291.23132, 95.08588, 81.07042
95	19.64	Oleoyl ethanolamide	$C_{20}H_{39}NO_2$	$[M+H-H_2O]^+$	325.2981	308.2939	6.1482	72.04504, 114.09147, 55.05509
96	20.17	Stearamide	$C_{18}H_{37}NO$	$[M+H]^+$	284.2937	283.2875	-3.8692	57.07079, 72.04510, 116.10714
97	20.61	Bis(2-ethylhexyl) phthalate	$C_{24}H_{38}O_4$	$[M+H]^+$	391.2826	390.277	-4.3447	149.02307, 167.03362, 93.03397
98	20.71	Tridemorph	$C_{19}H_{39}NO$	$[M+H]^+$	298.3096	297.3032	-3.0170	102.09167, 57.07076, 116.10715
99	20.71	Bis(2-ethylhexyl)adipate	$C_{22}H_{42}O_4$	$[M+H]^+$	371.3144	370.3083	-3.2318	147.06482, 101.05994, 129.05447
100	20.72	Linolenic acid ethyl ester	$C_{20}H_{34}O_2$	$[M+H]^+$	307.2622	306.2559	-3.2545	81.07040, 67.05492, 95.08587
101	22.50	Docosanamide	$C_{22}H_{45}NO$	$[M+H]^+$	340.3565	339.3501	-2.6443	284.29425, 57.07073, 88.07623

102	25.35	Triphenyl phosphate	$C_{18}H_{15}O_4P$	$[M+H]^+$	327.0768	326.0708	-3.9746	152.06180, 233.03566, 95.04954

Table S2-2 Components identified from EAEPC by UPLC-Q-TOF-MS/MS under negative ion mode.

No.	t_R (min)	Identification	Elemental compoisition	Proposed Ions	Experimental Mass (m/z)	Theoretical Mass (m/z)	Mass Error (ppm)	MS/MS(m/z)
1	0.98	Citric acid	$C_6H_6O_6$	$[M-H]^-$	191.0187	192.0270	-5.2351	111.00732, 87.00722, 85.02796
2	1.16	Uridine	$C_9H_{12}N_2O_6$	$[M-H]^-$	243.0619	244.0695	-1.2343	110.02330, 200.05559, 82.02827
3	1.97	4-Pyridoxic acid	$C_8H_9NO_4$	$[M-H]^-$	182.0448	183.0532	-6.0424	138.05475, 108.04403, 182.04482
4	3.35	Xanthosine	$C_{10}H_{12}N_4O_6$	$[M-H]^-$	283.0683	284.0757	-0.3533	151.02487, 108.01885
5	3.89	DL-Tryptophan	$C_{11}H_{12}N_2O_2$	$[M-H]^-$	203.0817	204.0899	-4.4317	116.04913, 74.02319, 142.06493
6	4.20	N-Acetylvaline	$C_7H_{13}NO_3$	$[M-H]^-$	158.0810	159.0895	-7.5910	116.07024, 114.09098
7	5.57	2-Isopropylmalic acid	$C_7H_{12}O_5$	$[M-H]^-$	175.0600	176.0674	-0.5712	115.03857, 85.06430, 113.05933
8	5.83	3,4-Dihydroxyphenylpropionic acid	$C_9H_{10}O_4$	$[M-H]^-$	181.0497	182.0579	-4.9710	59.01228, 137.05945, 119.04880
9	6.36	Vanillic acid	$C_8H_8O_4$	$[M-H]^-$	167.0339	168.0423	-6.5854	152.01028, 108.02021, 123.04378
10	6.34	2,2-Dimethylglutaric acid	$C_7H_{12}O_4$	$[M-H]^-$	159.0650	160.0736	-8.1727	115.07501,97.06438
11	6.59	Caffeic acid	$C_9H_8O_4$	$[M-H]^-$	179.0338	180.0423	-6.7026	135.04379, 134.03600
12	6.68	Pimelic acid	$C_7H_{12}O_4$	$[M-H]^-$	159.0650	160.0736	-8.1727	97.06435, 115.07499, 95.04872
13	7.23	N-Acetyl-D-alloisoleucine	$C_8H_{15}NO_3$	$[M-H]^-$	172.0967	173.1052	-6.9728	130.08594, 128.10674
14	7.65	3-Phenyllactic acid	$C_9H_{10}O_3$	$[M-H]^-$	165.0545	166.0630	-7.2703	147.04384, 119.04880, 72.99152
15	8.23	N-Acetyl-L-phenylalanine	$C_{11}H_{13}NO_3$	$[M-H]^-$	206.0813	207.0895	-4.3672	164.07050, 147.04387, 58.02826
16	8.67	Ferulic acid	$C_{10}H_{10}O_4$	$[M-H]^-$	193.0498	194.0579	-4.1440	134.03593, 178.02606, 193.04964

17	8.78	Suberic acid	$C_8H_{14}O_4$	$[M-H]^-$	173.0808	174.0892	-6.3554	111.08004, 83.04866, 129.09071
18	9.05	Lariciresinol 4-O-glucoside	$C_{26}H_{34}O_{11}$	$[M-H]^-$	521.2023	522.2101	-0.9593	329.13913, 160.05171, 175.07535
19	9.44	Rutin	$C_{27}H_{30}O_{16}$	$[M-H]^-$	609.1454	610.1519	1.3133	301.03513, 151.00241
20	10.07	Quercetin-3β-D-glucoside	$C_{21}H_{20}O_{12}$	$[M-H]^-$	463.0880	464.0955	-0.4319	300.02731, 271.02463, 301.03513
21	10.48	Azelaic acid	$C_9H_{16}O_4$	$[M-H]^-$	187.0965	188.1049	-5.8793	125.09579, 97.06436, 169.08580
22	10.78	Astragalin	$C_{21}H_{20}O_{11}$	$[M-H]^-$	447.0927	448.1006	-1.3420	255.02943, 284.03247, 227.03423
23	11.73	Quercetin	$C_{15}H_{10}O_7$	$[M-H]^-$	301.0349	302.0427	-1.6609	151.00238, 121.02806, 178.99751
24	12.10	Luteolin	$C_{15}H_{10}O_6$	$[M-H]^-$	285.0403	286.0477	-0.3508	133.02811, 151.00241, 175.03897
25	13.57	Corchorifatty acid F	$C_{18}H_{32}O_5$	$[M-H]^-$	327.2171	328.2250	-1.8336	211.13313, 229.14378, 171.10152
26	14.07	Dodecanedioic acid	$C_{12}H_{22}O_4$	$[M-H]^-$	229.1438	230.1513	-0.8728	221.13316, 167.14297
27	14.11	(15Z)-9,12,13-Trihydroxy-15-octadecenoic acid	$C_{18}H_{34}O_5$	$[M-H]^-$	329.2328	330.2406	-1.5187	211.13313, 171.10155, 229.14381
28	17.04	9-Oxo-ODE	$C_{18}H_{30}O_3$	$[M-H]^-$	293.2121	294.2195	-0.3410	277.21628, 67.05501, 81.07053
29	18.98	2,2'-Methylenebis(4-methyl-6-tert-butylphenol)	$C_{23}H_{32}O_2$	$[M-H]^-$	339.2322	340.2402	-2.0635	163.11163
30	20.29	Palmitic acid	$C_{16}H_{32}O_2$	$[M-H]^-$	255.2325	256.2402	-1.5672	59.01224, 90.08094, 169.13289
31	20.60	trans-Petroselinic acid	$C_{18}H_{34}O_2$	$[M-H]^-$	281.2484	282.2559	-0.7111	96.95870, 59.01220
32	21.43	Stearic acid	$C_{18}H_{36}O_2$	$[M-H]^-$	283.2640	284.2715	-0.7061	265.25461, 193.76231

Table S3 Topological freedom of compounds in EAEPC based on PPI networks

Compounds	Degree
Quercetin	207
Adenosine	161
Guanosine	156
Kaempferol	124
Palmitic acid	98
Arachidonic acid	74
Luteolin	73
Esculetin	73
Azelaic acid	49
Uridine	48
Cinnamaldehyde	42
Citric acid	39
Betaine	38
Syringic acid	36
Bis(2-ethylhexyl)adipate	31
Astragalin	25
Coumarin	24
Choline	23
Citroflex 4	21
L-Norleucine	19
Caffeic acid	19
Bis(2-ethylhexyl)phthalate	19
2,4-Quinolinediol	19
L-Isoleucine	19
Vanillic acid	18
Quinoline	18
Phenethylamine	18
N-Acetyl-L-Phenylalanine	18
N-Acetyl-DL-tryptophan	18
Mesterolone	18
Dibutyl phthalate	18
Ambrosic acid	18
8-Hydroxyquinoline	18
3-Methoxybenzaldehyde	18
2,2'-Methylenebis(4-methyl-6-tert-butylphenol)	18
L-Pyroglutamic acid	17
Isoleucine	17
Indole-3-acrylic acid	17
9-Oxo-ODE	17
4-Phenylbutyric acid	17
2-Isopropylmalic acid	17
2,2-Dimethylglutaric acid	17
1,5-Isoquinolinediol	17
L-Histidine	17
Xanthurenic acid	16
Xanthosine	16
Tetranor-12R-HETE	16
Stearamide	16
Sinapinic acid	16
Sinapine	16
Salicylic acid	16
Oleoyl ethanolamide	16
N-Acetylvaline	16
N-Acetyltyramine	16

N-Acetyl-L-leucine	16
N-Acetyldopamine	16
N-Acetyl-D-alloIsoleucine	16
Methenolone	16
Matairesinol	16
L-Phenylalanine	16
Linoleoyl ethanolamide	16
Isophorone	16
Indole	16
Hexadecanamide	16
Ferulic acid	16
Dodecanedioic acid	16
Docosanamide	16
DL-Tryptophan	16
Desthiobiotin	16
Corchorifatty acid F	16
cis,cis-Muconic acid	16
Caprolactam	16
BMK methyl glycidate	16
Ageratriol	16
Afzelin	16
5-Hydroxyindole-3-acetic acid	16
5-Ethylcyclohexane-1,3-dione	16
3,4-Dihydroxyphenylpropionic acid	16
2-Hydroxycinnamic acid	16
1-Methylguanine	16
19-Norandrostenedione	16
13,14-Dihydro prostaglandin E1	16
12-Oxo phytodienoic acid	16
10-HAD	16
(9cis)-Retinal	16
(15Z)-9,12,13-Trihydroxy-15-octadecenoic acid	16
(+/-)-C75	16
Valylproline	16
Pimelic acid	16
Tridemorph	16
Porphyrin	16
Sinapic acid	16
Proline	15
Adenine	15
6-Quinolinecarboxylic acid	15
trans-Cinnamaldehyde	14
Lariciresinol 4-O-glucoside	13
3-Phenyllactic acid	12
Guanine	11
Acetophenone	11
6-Methylquinoline	11
4-Indolecarbaldehyde	11
Suberic acid	10
Rutin	9
DL-Stachydrine	9
Trigonelline	8
D-Glucosamine	7
4-Aminobenzoic acid	6
Nicotinic acid	5
4-Pyridoxic acid	5
3-Aminophenol	5

Anthranilic acid	4
Linolenic acid ethyl ester	3
DL-Arginine	3
Cytosine	3
Stearic acid	2
Quercitrin	2
Pyridoxine	2
Dodecamethylcyclohexasiloxane	2
1,5-Naphthalenediamine	2
Thymine	2

Table S4 MRM conditions and mass spectral parameters for major active ingredients

Compounds	Precursor ion (m/z)	Product ion (m/z)	CE （ev）	t_R (min)	MRM scanning mode
Quercetin	303.0600	153.0100	32	15.940	Positive
Adenosine	268.1000	136.0600	15	10.420	Positive
Guanosine	284.1000	152.0500	9	10.535	Positive
Kaempferol	287.0500	153.0100	34	17.330	Positive
Palmitic acid	255.2300	237.2200	21	9.612	Negative
Arachidonic acid	305.2400	119.0800	32	26.283	Positive
Luteolin	179.0300	123.0400	22	12.960	Positive
Esculetin	287.0500	153.0100	34	16.303	Positive
Azelaic acid	187.0900	125.0900	15	6.392	Negative
Uridine	245.0700	113.0300	8	10.415	Positive
